# Diagnostic Complexities and Management of Gouty Arthritis With Suspected Seronegative Rheumatoid Arthritis and Malignancy in an Elderly Male

**DOI:** 10.7759/cureus.68860

**Published:** 2024-09-07

**Authors:** Sai Pritam Keelapattu, Ajay Bangaragiri, Chaitanya Sai Eada

**Affiliations:** 1 Medicine and Surgery, North Cumbria Integrated Care NHS Foundation Trust, Carlisle, GBR; 2 Radiology, North Cumbria Integrated Care NHS Foundation Trust, Carlisle, GBR; 3 General Surgery, North Cumbria Integrated Care NHS Foundation Trust, Carlisle, GBR

**Keywords:** arthritis management, autoimmune synovitis, chronic gout, diagnostic challenges, imaging in arthritis, knee pain, mri, rheumatoid arthritis, seronegative ra, synovial hypertrophy

## Abstract

Rheumatoid arthritis (RA) and gout are two distinct types of inflammatory arthritis with significant morbidity. While RA is characterized by autoimmune synovitis, gout is defined by the deposition of urate crystals. Diagnosing these conditions becomes particularly challenging in patients with negative serological markers for RA, compounded by the patient's advanced age and potential for malignancy. This case involves a 77-year-old male with chronic gout, hypertension, chronic atrial fibrillation on edoxaban, diastolic congestive heart failure, and chronic kidney disease stage 3B, presenting with left knee pain and limited mobility. Despite negative serology for RA (rheumatoid factor (RF) <20.0 IU/ml, anti-CCP2 antibodies 1.2 U/mL), the clinical presentation raised suspicion for RA. Imaging revealed significant synovial hypertrophy and multiple periarticular lesions suggestive of chronic gouty tophi rather than RA or malignancy. The patient was managed with allopurinol, prednisolone, and colchicine and referred to rheumatology for further evaluation. Approximately 30% of RA patients may present with negative serological markers, complicating the diagnosis. Differentiating RA from gout is crucial due to differences in management strategies. Imaging modalities such as MRI and CT are essential in identifying characteristic changes of both conditions, such as synovial hypertrophy in RA and tophi in gout. In elderly patients, the possibility of malignancy should also be considered. This case highlights the complexity of diagnosing gouty arthritis mimicking seronegative RA, especially in elderly patients where the risk of malignancy must be considered. It underscores the need for comprehensive clinical and imaging evaluations and personalized treatment plans in managing patients with multiple comorbidities.

## Introduction

Gout, an inflammatory arthritis, results from the deposition of monosodium urate crystals in the joints and soft tissues. It primarily affects men over the age of 40 with comorbidities such as obesity, hypertension, and chronic kidney disease predisposing to its development. The hallmark clinical manifestation is acute monoarthritis, commonly involving the first metatarsophalangeal joint, known as podagra [1]. Diet and genetic polymorphisms of renal transporters of urate seem to be the main causal factors of primary gout [2]. Chronic gout can lead to the formation of tophi, which can mimic other pathological conditions such as rheumatoid arthritis (RA) and neoplastic processes in imaging studies.

Rheumatoid arthritis is another form of chronic inflammatory disorder primarily affecting the joints [3]. It is typically diagnosed through clinical examination and serological markers such as rheumatoid factor (RF) and anti-cyclic citrullinated peptide (anti-CCP) antibodies. However, approximately 30% of RA patients may present with negative serology, complicating the diagnostic process.

The coexistence of gout and RA is rare but can present significant diagnostic and therapeutic challenges. This report describes a case of an elderly male with chronic gout and suspected RA, with a focus on the diagnostic complexities and management strategies employed, including the consideration of potential malignancy. The objective is to illustrate the importance of a comprehensive diagnostic approach in patients with overlapping clinical features of multiple arthritic conditions.

## Case presentation

A 77-year-old male with a history of chronic gout, hypertension, chronic atrial fibrillation, congestive heart failure, and chronic kidney disease was admitted following a fall. The patient reported severe left knee pain and limited mobility.

On physical examination, he had tophi on multiple fingers and elbows and a significant body mass index (BMI) of 42.120 kg/m^2^. His lab results showed negative RA serology with normal parameters of RF <20.0 IU/ml (normal reference range: 0-20 IU/ml), anti-CCP2 antibodies 1.2 U/ml (normal reference: 0-10 U/mL), and raised parameters of C-reactive protein (CRP) 60 mg/L (normal reference range: 0-5 mg/L), erythrocyte sedimentation rate (ESR) 36 mm/hr (normal reference range: 0-15 mm/hr), urea acid 640 umol/L (normal reference range: 200-430 umol/L), and a negative screen for human leukocyte antigen B27 test (HLA B27).

**Figure 1:**
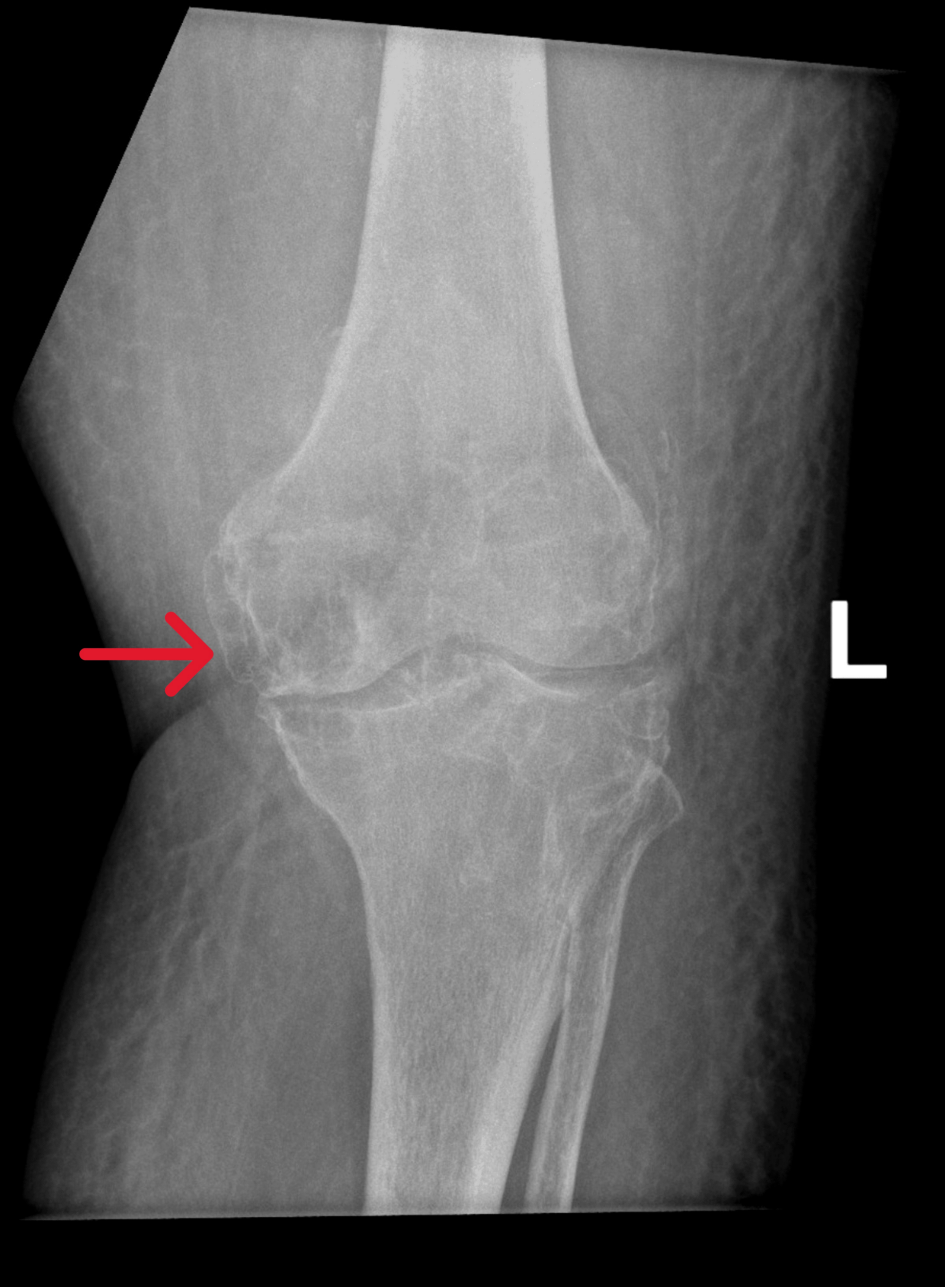
An X-ray (AP view) demonstrates subarticular radiolucencies in the medial aspect of the distal femur AP view: anterior-posterior view

**Figure 2:**
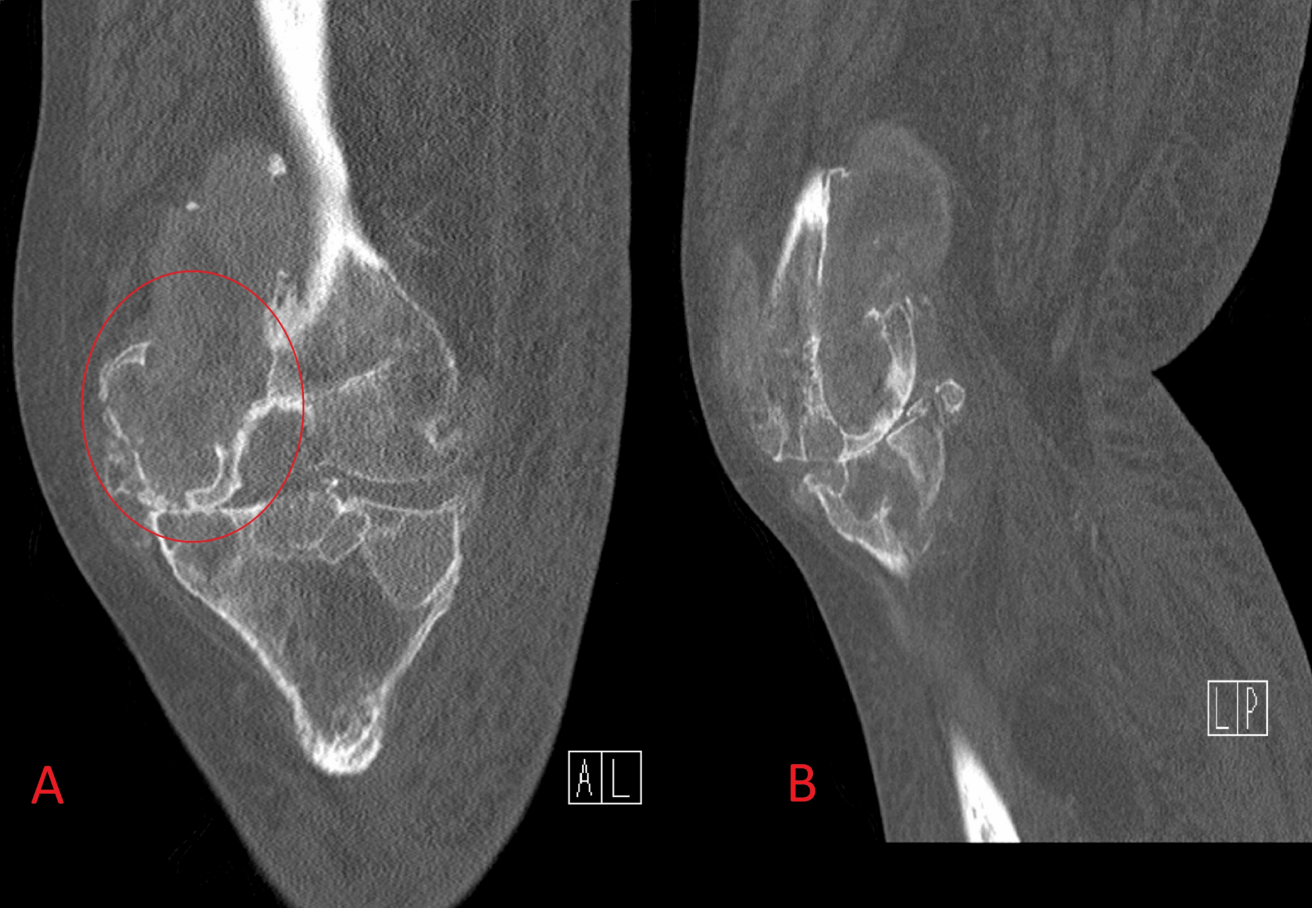
The CT scan demonstrates subcortical cysts with intra-articular soft tissue extensions and extensive cortical destruction with decreased joint space. 2A: coronal reconstructed image; 2B: sagittal reconstructed image

**Figure 3:**
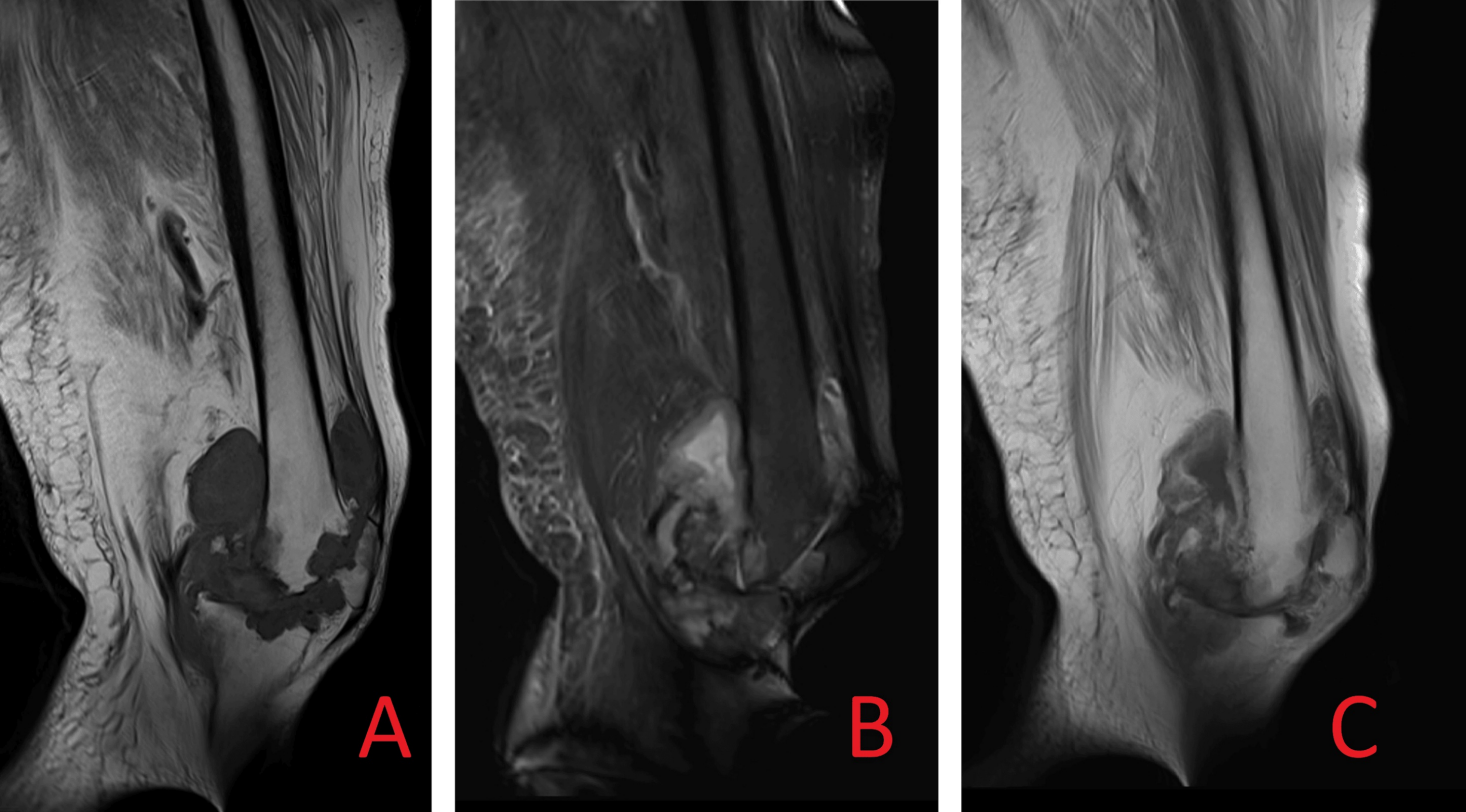
The MRI scans demonstrate irregular areas of soft tissue signal intensity, which are T1 hypointense and PDFS hyperintense, showing post-contrast enhancement seen extending into joint space. 3A: T1-weighted MRI; 3B: T2-weighted MRI; 3C: post-contrast MRI PDFS: proton density fat-suppressed

X-ray (Figure 1), CT scan (Figure 2), and MRIs (Figure 3) of the left knee revealed subarticular radiolucencies, peripheral enhancement, central necrosis, and subcortical cysts, respectively.

The patient was initially managed with allopurinol 50 mg, increasing every four weeks to a maximum of 200 mg, colchicine 500 micrograms twice daily, adjusted according to his renal function, and a short course of prednisolone. He was also prescribed lansoprazole to mitigate gastrointestinal side effects. The patient was referred to rheumatology for further evaluation and ongoing management.

## Discussion

This case underscores the diagnostic challenges presented by the coexistence of gout and RA, especially in seronegative cases. Gout presents a diagnostic challenge in elderly patients with multiple comorbidities, often mimicking both clinically and radiologically other forms of inflammatory arthritis such as RA [4]. Negative serological markers (RF and anti-CCP antibodies) and atypical joint involvement patterns further complicate the diagnosis. Imaging findings are critical in differentiating these conditions, showing chronic synovial hypertrophy, multiple peri-articular lesions with varying degrees of calcification, and significant degenerative changes, suggestive of gout rather than RA or malignancy.

In the literature, the coexistence of gout and RA is rare, with only a few documented cases. Chronic gouty arthritis, particularly in patients with long-standing hyperuricemia, can lead to the formation of tophi within the synovium, which can mimic the pannus formation seen in RA. The peri-articular lesions and synovial hypertrophy observed in this patient were suggestive of gouty tophi rather than RA. This differentiation is critical, as the treatment approaches for gout and RA differ significantly. While disease-modifying anti-rheumatic drugs (DMARDs) are central to RA management, gout is primarily managed through urate-lowering therapies and anti-inflammatory medications during acute flares [5,6].

The possibility of malignancy such as osteosarcoma was also considered in this case due to the patient's advanced age and the imaging findings of calcified peri-articular lesions. In elderly patients, particularly those with chronic inflammatory conditions, the differential diagnosis should include malignancy, especially when there is a new onset of symptoms or imaging findings that are atypical for the known condition. In this case, the lack of systemic symptoms typically associated with malignancy (such as unexplained weight loss, night sweats, or significant lymphadenopathy) and the stable nature of the lesions over time helped to rule out the neoplastic process.

The patient was initially managed with a combination of pharmacological therapies targeting both gout and RA. The treatment regimen began with allopurinol, a xanthine oxidase inhibitor, starting at 50 mg daily. This dose was titrated up every four weeks to a maximum of 200 mg daily, and colchicine was prescribed at 500 micrograms twice daily as a prophylactic agent to prevent acute gout attacks during the urate-lowering phase [7]. The patient also received a short course of prednisolone to address the acute inflammation and pain, a common practice for managing acute flare-ups of gouty arthritis. Prednisolone, at a dosage of 20 mg daily, was tapered over a two-week period. Lansoprazole was prescribed to mitigate the gastrointestinal side effects of both prednisolone and colchicine, which are particularly concerning in elderly patients.

Follow-up

Given the patient’s age and the chronic nature of his condition, there was a significant concern regarding the long-term management of his symptoms and the prevention of further complications. Regular follow-up was essential to monitor the patient's response to therapy, adjust medication dosages, and manage any adverse effects.

The patient was referred to rheumatology, and their evaluation ultimately supported the diagnosis of gouty arthritis mimicking seronegative RA, with no definitive evidence of malignancy found on further imaging. This conclusion was based on the chronicity of the tophi, the pattern of joint involvement, and the response to gout-specific therapy. Despite the absence of malignancy, the patient's age necessitated ongoing vigilance for any new symptoms that could indicate a neoplastic process.

Management of patients with both gout and RA necessitates a customized treatment plan that takes into account the patient's comorbidities, often requiring interdisciplinary collaboration to achieve the best possible outcomes [4]. A multidisciplinary approach with collaboration between primary care physicians, rheumatologists, radiologists, and potentially oncologists is vital to ensure that all potential diagnoses are thoroughly evaluated and that treatment plans are both comprehensive and individualized. This approach is critical in mitigating the risk of medication-related adverse effects and ensuring effective disease control.

## Conclusions

This case illustrates the diagnostic and therapeutic challenges posed by gouty arthritis mimicking seronegative RA, especially in elderly patients at increased risk of malignancy. Despite negative serological markers, the patient's clinical presentation and imaging findings necessitated a comprehensive diagnostic approach. Chronic gout can mimic RA both clinically and radiologically, complicating accurate diagnosis and management. Increased reporting of similar cases can enhance understanding and awareness of the overlapping features of these conditions. Regular discussion and publication of such cases will help physicians recognize and consider the possibility of coexisting RA and gout, even in seronegative patients. Further research and clinical studies are needed to establish more effective diagnostic markers and tailored treatment strategies for managing patients with multiple arthritic conditions, especially those with significant comorbidities. Ultimately, a multidisciplinary approach remains crucial in optimizing patient outcomes in complex cases like this.
